# Supplementary figures and images for: Spatial Heterogeneity in Bistable Figure-Ground Perception
Source: Iperception. 2020 Oct 20;11(5):2041669520961120. doi: 10.1177/2041669520961120 (PMC7594238; doi:10.1177/2041669520961120)

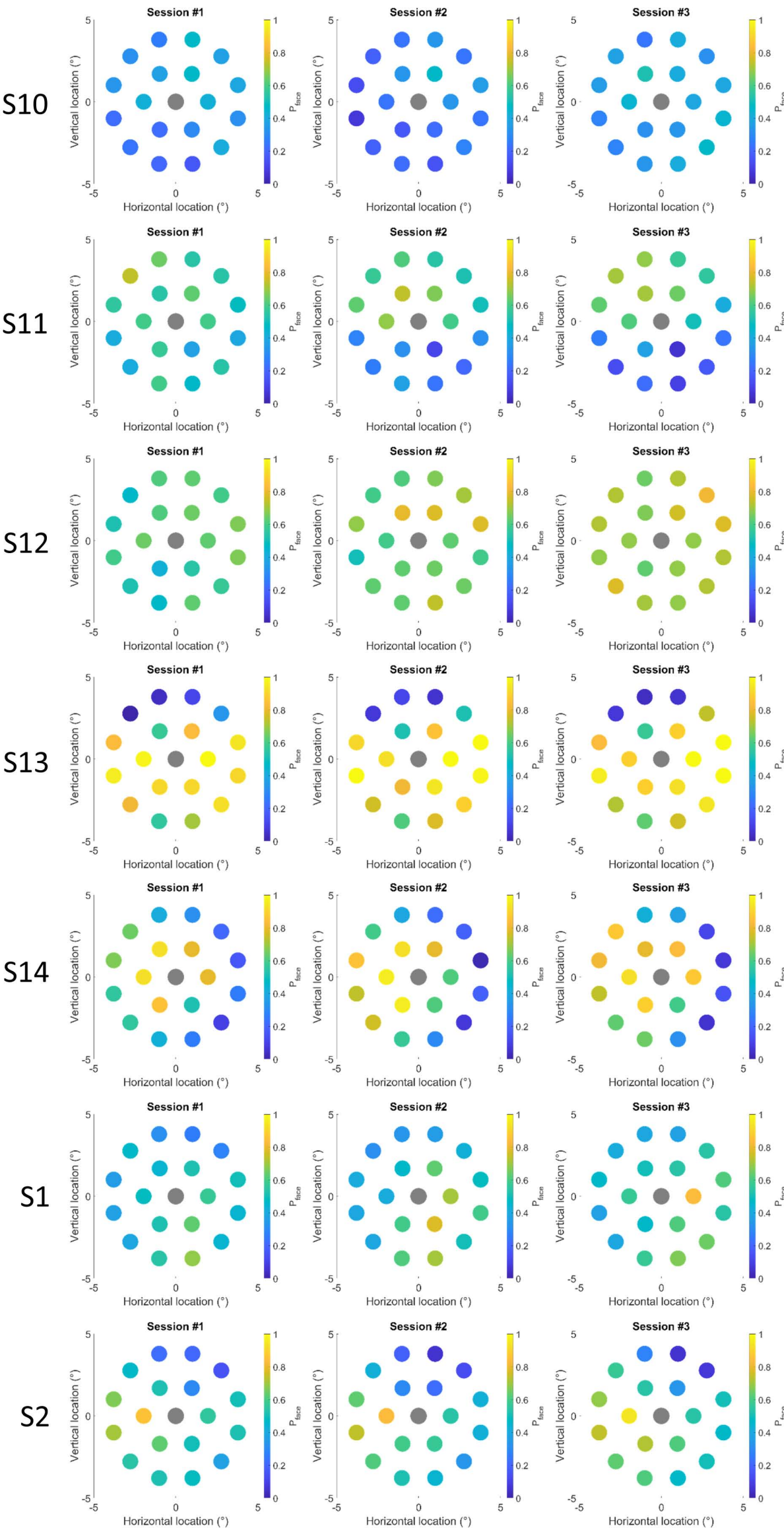

Supplement: sj-pdf-1-ipe-10.1177_2041669520961120 - Supplemental material for Spatial Heterogeneity in Bistable Figure-Ground Perception [file sj-pdf-1-ipe-10.1177_2041669520961120.pdf]

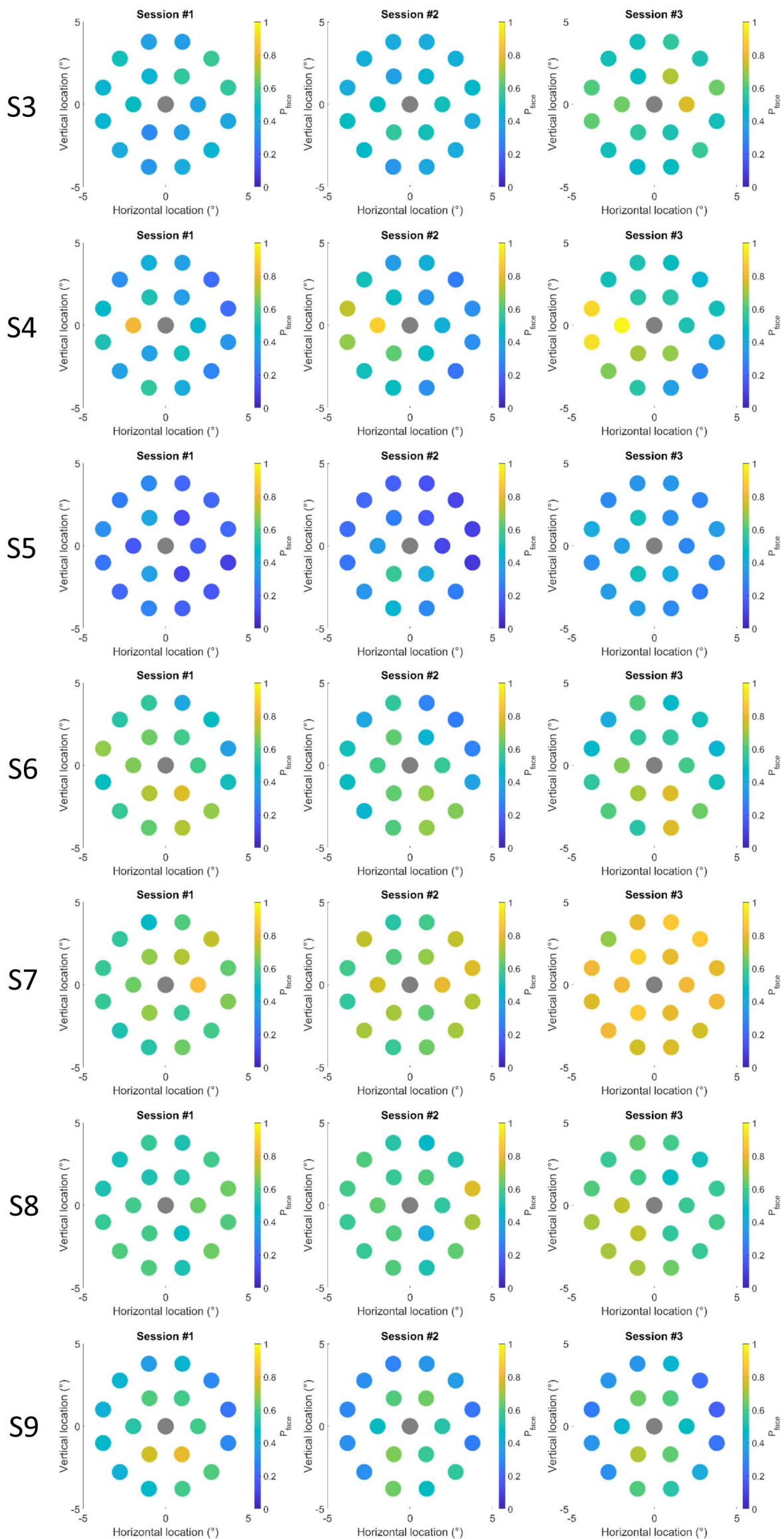

Supplement: sj-pdf-2-ipe-10.1177_2041669520961120 - Supplemental material for Spatial Heterogeneity in Bistable Figure-Ground Perception [file sj-pdf-2-ipe-10.1177_2041669520961120.pdf]

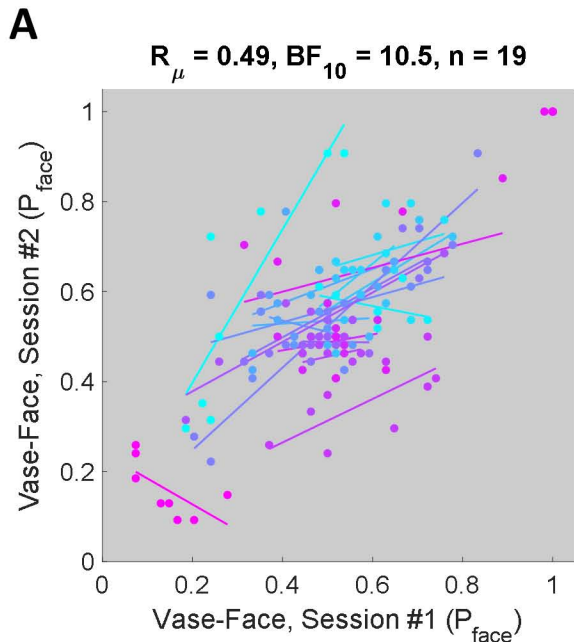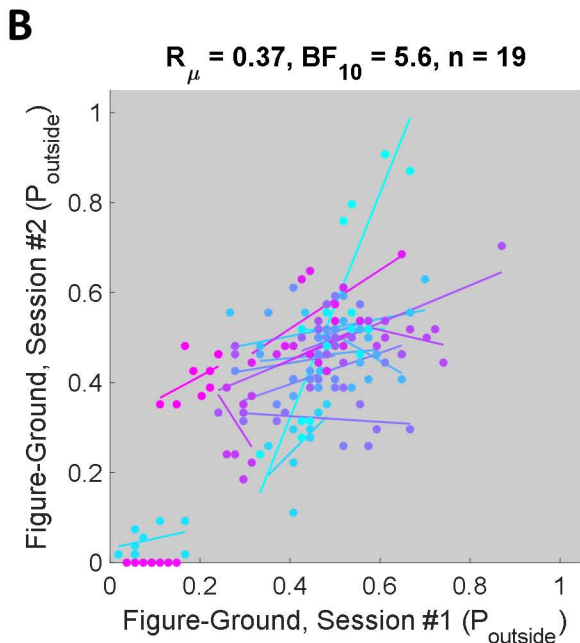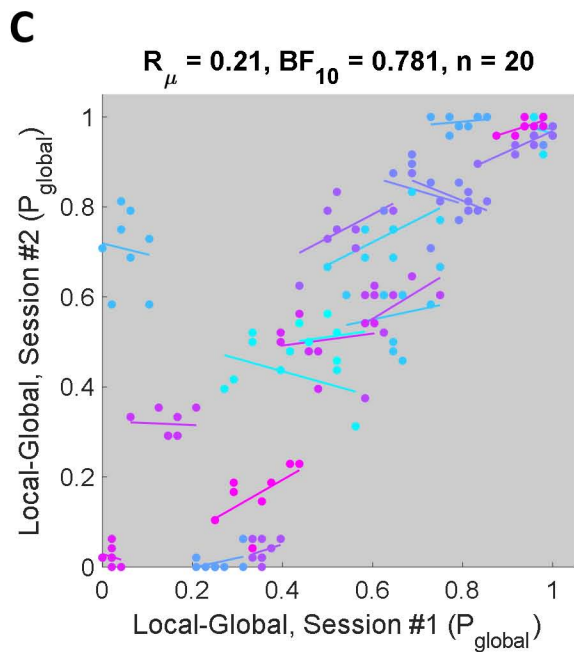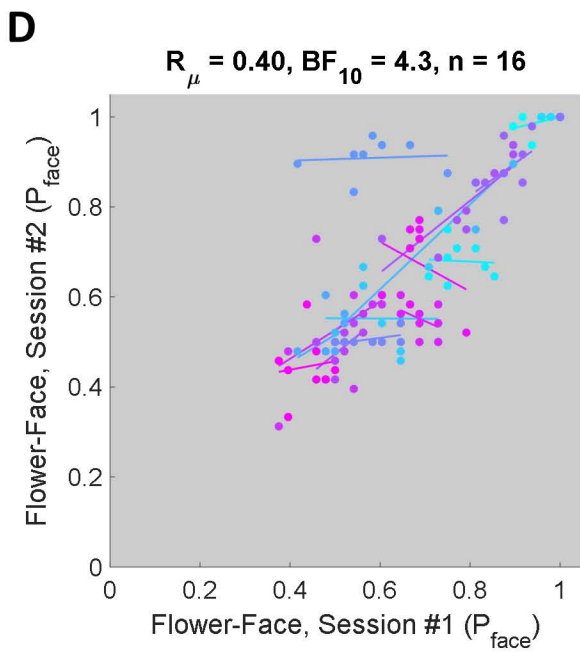

Supplement: sj-pdf-3-ipe-10.1177_2041669520961120 - Supplemental material for Spatial Heterogeneity in Bistable Figure-Ground Perception [file sj-pdf-3-ipe-10.1177_2041669520961120.pdf]
