# Supplementary material for: Spatial Heterogeneity in Bistable Figure-Ground Perception
Source: Iperception. 2020 Oct 20;11(5):2041669520961120. doi: 10.1177/2041669520961120 (PMC7594238; doi:10.1177/2041669520961120)
Supplement: sj-pdf-4-ipe-10.1177_2041669520961120 - Supplemental material for Spatial Heterogeneity in Bistable Figure-Ground Perception [file sj-pdf-4-ipe-10.1177_2041669520961120.pdf]

Spatial heterogeneity in bistable figure-ground perception

Nonie J. Finlayson, Victorita Neacsu, & D. Samuel Schwarzkopf

Supplementary Information

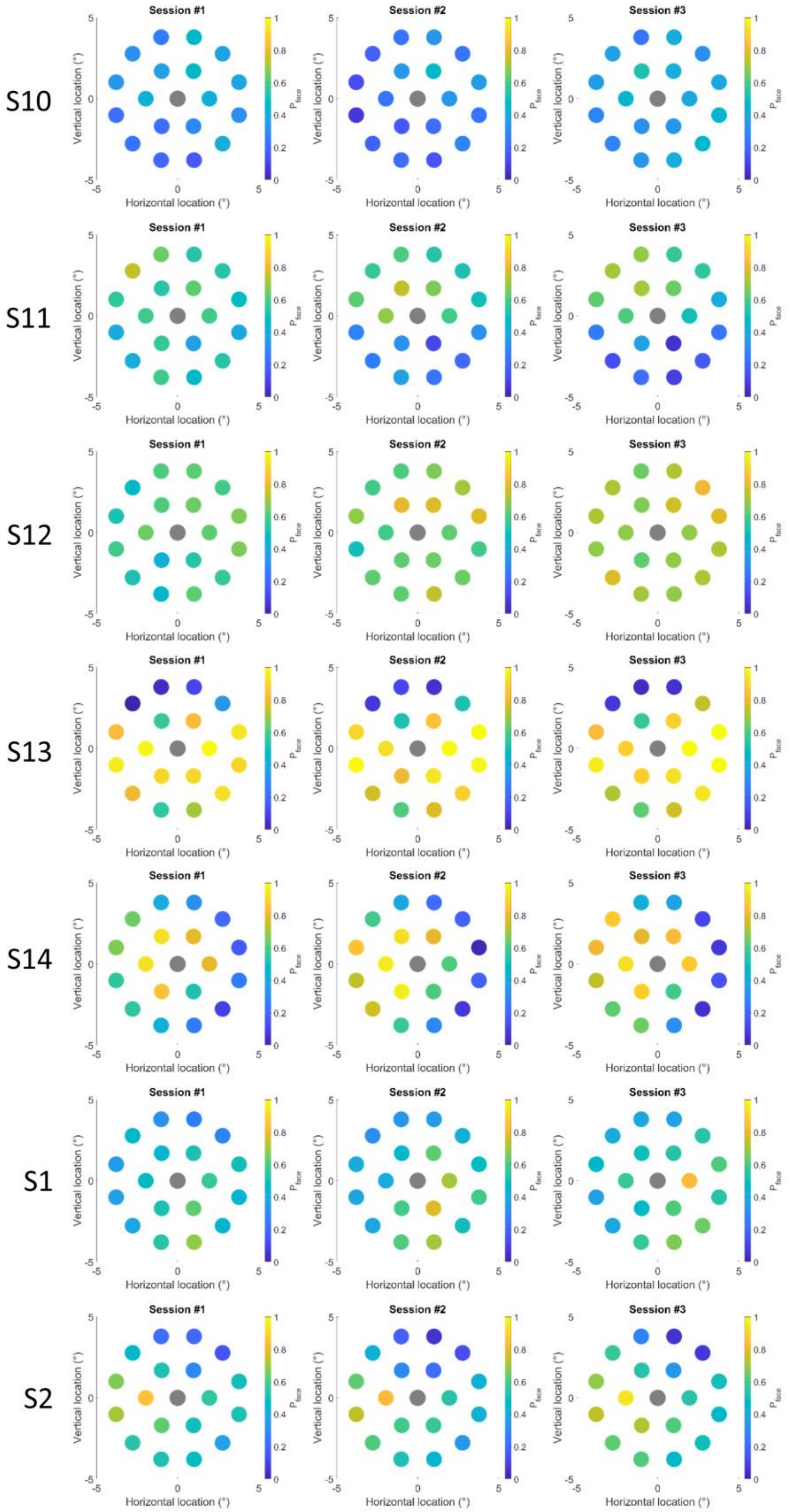

Supplementary Figure S1. Individual data from 7 participants in Experiment 1. The proportion of trials the participant reported seeing faces (see color code) in each session plotted at each visual field location tested. Each row shows data from a different participant. (The grey dot at fixation is a placeholder).

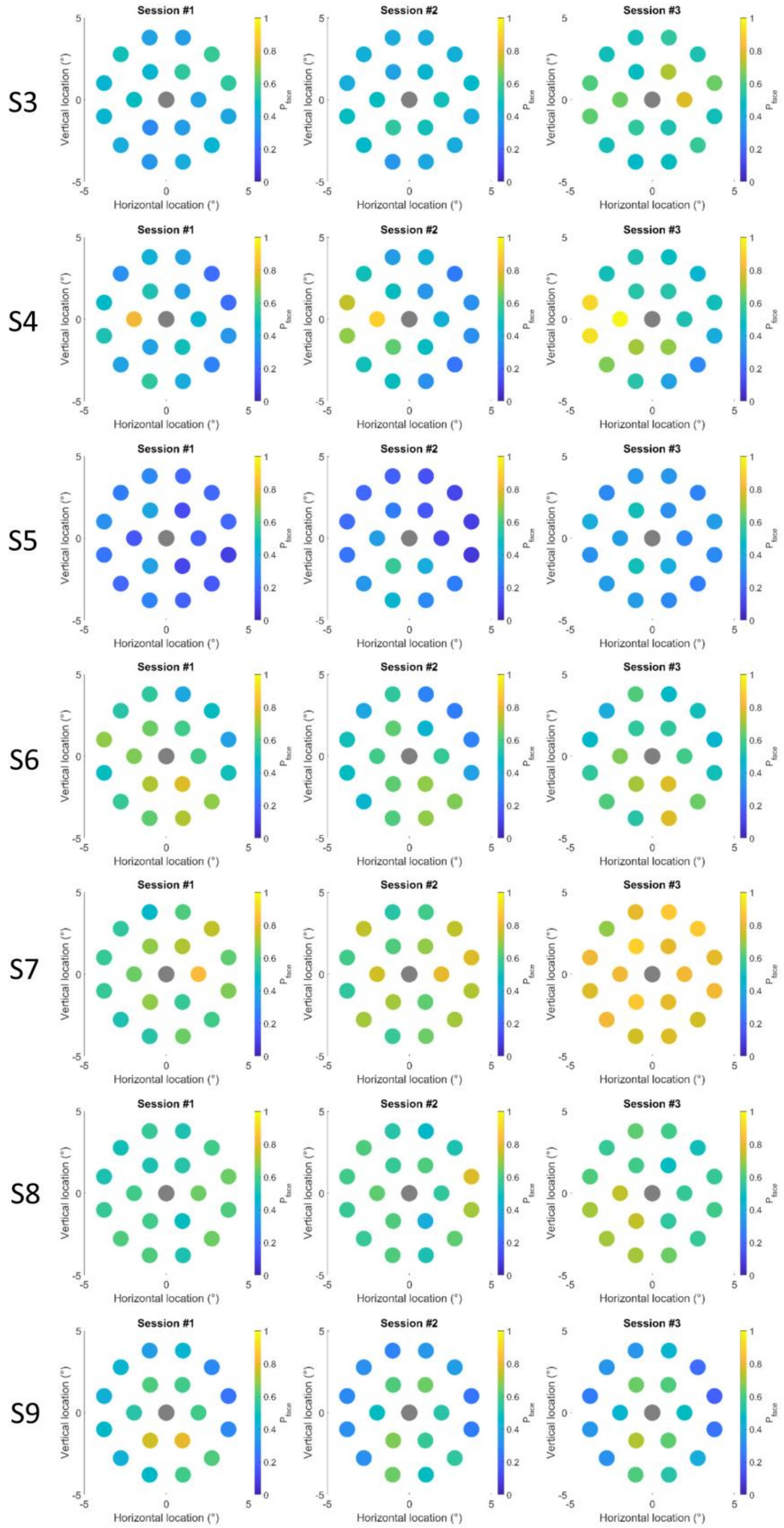

Supplementary Figure S2. Individual data from the other 7 participants in Experiment 1. The proportion of trials the participant reported seeing faces (see color code) in each session plotted at each visual field location tested. Each row shows data from a different participant. (The grey dot at fixation is a placeholder).

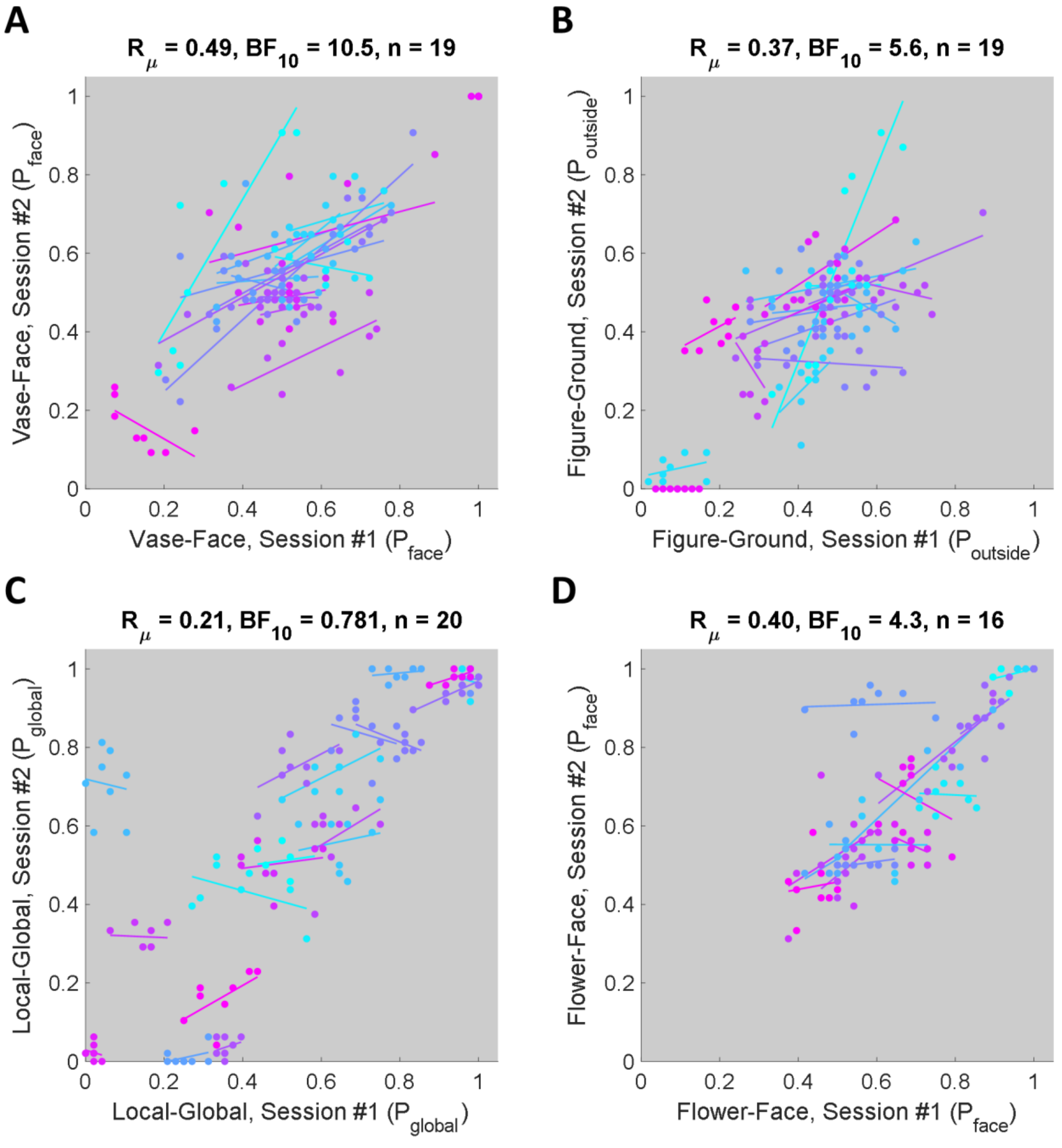

Supplementary Figure S3. Intersession correlation of bias patterns in Experiments 2-5. Data in session 2 were plotted against session 1 across all participants. Dots denote for a given location the proportion of trials participants reported, respectively, seeing faces (A and D), the outer part of inverted vase-face images as figure (B), or the global configuration in Navon-like stimuli (C). Different colors indicate individual participants. Solid lines show a linear regression between sessions across locations for each participant.
