# Supplementary material for: Spatial Heterogeneity in Bistable Figure-Ground Perception
Source: Iperception. 2020 Oct 20;11(5):2041669520961120. doi: 10.1177/2041669520961120 (PMC7594238; doi:10.1177/2041669520961120)
Supplement: sj-pdf-5-ipe-10.1177_2041669520961120 - Supplemental material for Spatial Heterogeneity in Bistable Figure-Ground Perception [file sj-pdf-5-ipe-10.1177_2041669520961120.pdf]

Spatial heterogeneity in bistable figure-ground perception

*Nonie J. Finlayson [1,2], Victorita Neacsu [3], & D. Samuel Schwarzkopf [1,4 *]*

1. Experimental Psychology, University College London, U.K.
2. Ipsos Public Affairs, Brisbane, Australia
3. Wellcome Centre for Human Neuroimaging, University College London, U.K.
4. School of Optometry & Vision Science, University of Auckland, New Zealand

* To whom correspondence should be directed

D. Samuel Schwarzkopf
School of Optometry & Vision Science
Faculty of Medical & Health Sciences
University of Auckland
85 Park Road, Grafton
Auckland, New Zealand

Email: s.schwarzkopf@auckland.ac.nz

Abstract

The appearance of visual objects varies substantially across the visual field. Could such spatial heterogeneity be due to undersampling of the visual field by neurons selective for stimulus categories? Here we show that which parts of a bistable vase-face image observers perceive as figure and ground depends on the retinal location where the image appears. The spatial patterns of these perceptual biases were similar regardless of whether the images were upright or inverted. Undersampling by neurons tuned to an object class (e.g. faces) or variability in general local vs global processing cannot readily explain this spatial heterogeneity. Rather, these biases could result from idiosyncrasies in low-level sensitivity across the visual field.

Introduction

Our visual perception of the world is not homogeneous. Across individuals, visual acuity decreases from central to peripheral vision which accords with the fall-off in cortical magnification (Duncan & Boynton, 2003). Stimuli appear smaller in peripheral compared to central vision (Anstis, 1998; Bedell & Johnson, 1984; Helmholtz, 1867; Moutsiana et al., 2016; Newsome, 1972). Even basic visual features, such as object position, size, and shape, appear differently across the visual field and viewing conditions or when measured at different times (Afraz et al., 2010; Wexler, 2018; Wexler et al., 2015). Further studies confirmed such biases for the apparent size (Moutsiana et al., 2016; Schwarzkopf & Rees, 2013) and position (Kosovicheva & Whitney, 2017) of visual stimuli. A seminal study reported spatial heterogeneity in appearance for stimulus attributes ranging from size and orientation to the apparent gender or age of faces (Afraz et al., 2010). Crucially, while these bias patterns are reproducible across repeated tests, many of these bias patterns are unique to each individual.

The neural basis for this spatial heterogeneity still largely remains unknown. We showed that idiosyncratic biases in size perception correlate with the functional architecture and spatial selectivity of human V1 (Moutsiana et al., 2016; Schwarzkopf et al., 2011; Schwarzkopf & Rees, 2013). Specifically, we found that when population receptive fields are broader (less spatially selective) at a particular visual field location, observers perceive stimuli at this location as smaller. We argued that this is because the V1 response to a stimulus is more blurred when receptive fields are larger. In turn, later stages of visual processing then infer a smaller size because the representations of the stimulus edges are attracted to one another (Moutsiana et al., 2016). This could also explain consistent biases in perceived location: if a stimulus predominantly activates neurons with large receptive fields whose centers are located far away, this would result in a skewed activity profile that higher-order areas then read out as an incorrect location (Kosovicheva & Whitney, 2017). Some parts of the visual field are effectively undersampled by the receptive field mosaic producing errors in the population code for stimulus position.

Could a similar process underlie the spatial heterogeneity in perceiving complex attributes of objects, such as the gender of faces? Rather than only being tuned to position, neurons may be

selective for particular attributes like stimulus color or the gender of a face. Afraz and colleagues posited that if the receptive field mosaic undersamples the visual field, this could explain heterogeneity in perceiving facial identity or gender (Afraz et al., 2010; Visconti di Oleggio Castello et al., 2018). If a given visual field location is mostly covered by neurons most selective for female faces, an androgynous face image would predominantly activate these neurons and in turn the face appears more female.

Only limited evidence supports the existence of neurons specifically tuned to the gender of faces. Afraz and colleagues found that the degree of spatial heterogeneity in perceptual biases depends on stimulus size. There is little or no variability across the visual field if stimuli are too large. The critical size to observe heterogeneity is larger for more complex attributes like facial gender than simpler attributes like color (Afraz et al., 2010). This mirrors the presumed size of receptive fields and the mosaic density of neurons tuned to these attributes. Other evidence for gender or age-selective neurons comes from adaptation experiments (Hsu & Young, 2004; Schweinberger et al., 2010; Storrs & Arnold, 2012). However, some of these effects could be explained by lower-level adaptation. As such it remains uncertain if dedicated neurons for these complex stimulus attributes exist in the human visual system.

Here we set out to test the undersampling hypothesis using variations of Rubin's vase-face illusion, bistable images that can be perceived either as a vase or two faces in profile (Figure 2A). If more neurons sensitive to faces than vases cover a given retinal location, this should bias the percept towards seeing faces. Unlike for gender and age, it is relatively well-established that there are neuronal populations in the human visual system that are category-selective, such as the occipital and fusiform face areas, which respond preferentially to faces (e.g., O'Craven et al., 1999). Most of these regions retain at least some degree of retinotopic organization (Groen et al., 2017; Kay et al., 2015). Category-selective responses have also been measured in response to the vase-face illusion (U. Hasson et al., 2001; Peatfield et al., 2015). Therefore, we postulate that the receptive field mosaic in these regions might undersample parts of the visual field. An ambiguous vase-face stimulus might activate face-preferring neurons more in some locations than others, thus resulting in variations in perceptual judgments (Figure 1).

In line with this hypothesis, we indeed find reliable spatial heterogeneity for faces versus vases. We then tested whether these patterns are related to perceiving faces or other visual processes. We compared biases for perceiving faces in this bistable illusion to three other conditions: inverted versions of the same vase-face illusion, a different bistable illusion that could be interpreted as faces or plants, and a general task probing global versus local perceptual organization.

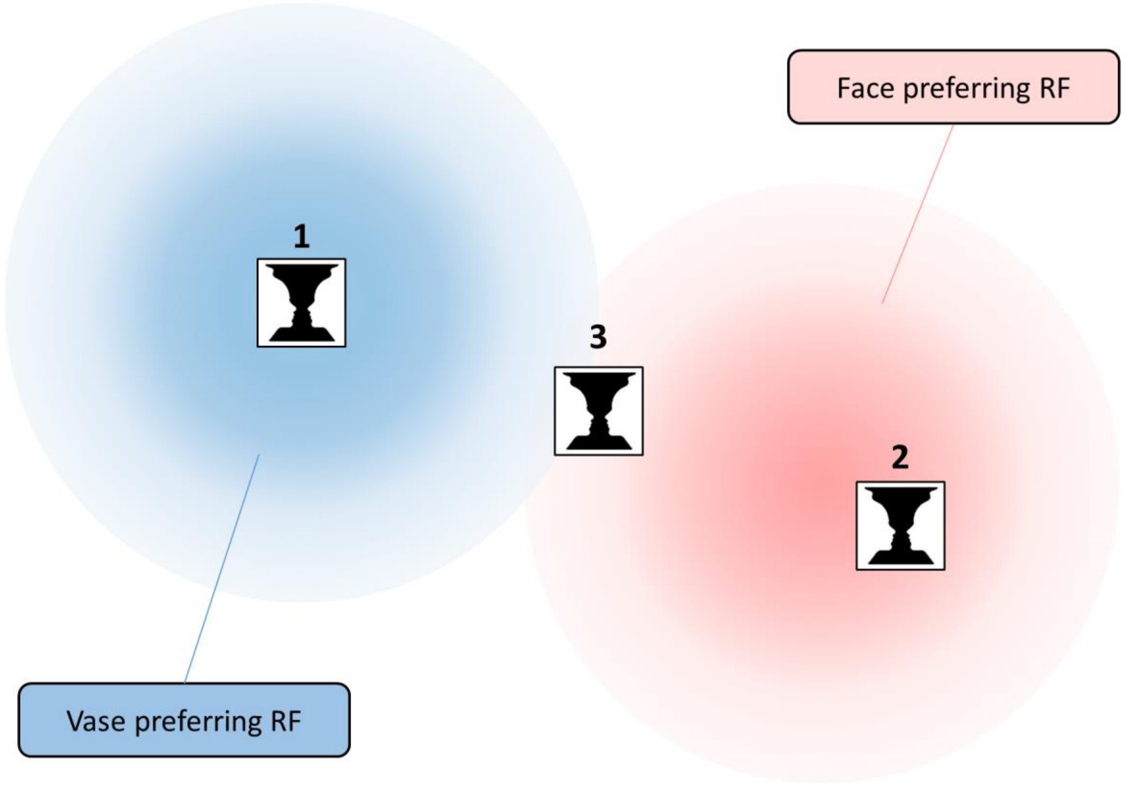

Figure 1. Illustration of the undersampling hypothesis. Consider two neurons with large receptive fields sparsely covering the visual field. One preferentially responds to vases (blue), the other to faces (red). A small ambiguous vase-face image presented at location 1 will almost exclusively activate the vase-preferring neuron and at location 2 the face-preferring neuron. Thus, the population code at those two locations would strongly favor the vase or face interpretation, respectively. In contrast, at location 3 both neurons will respond to the stimulus. However, it is still somewhat closer to the receptive field of the face-preferring neuron and thus the population code will be subtly biased towards faces.

Methods and Materials

Participants

Fourteen individuals (10 female, age range 21-38) took part in Experiment 1. Another sample of 20 individuals (9 female, age range 24-67) all participated in Experiments 2-5. The sample in Experiment 1 contained a number of practiced psychophysics observers, whereas we recruited participants more broadly for Experiments 2-5. Sample sizes were chosen based on previous similar work and upscaled somewhat (e.g. Afraz et al., 2010 tested 11 participants). Participants gave written informed consent and all procedures were approved by the University College London Research Ethics Committee.

All participants either had normal uncorrected visual acuity or were asked to wear their standard corrective lenses during the experiment. Because the age range for Experiments 2-5 was very wide, we repeated all analyses after removing three participants aged 45 and above. All findings from this analysis were extremely similar as the results reported here.

Stimuli
Participants viewed the stimuli at a distance of 48 cm, stabilized by a chin-and-forehead rest. Stimuli were presented on a Samsung 2233RZ liquid crystal display monitor with resolution 1680x1050 pixels and refresh rate of 120Hz. The minimum and maximum luminance was 0.25 and 230 cd m^{-2}, respectively. Stimuli were presented using MATLAB (The MathWorks Inc., Natick, MA) and the Psychtoolbox version 3 (Brainard, 1997; Pelli, 1997).

In Experiments 1 and 2, we used variations of the vase-face illusion (Figures 2A, 3A) harvested from an internet search of images in the public domain. They were further cropped and altered by one experimenter (VN) so that in the end there were 9 unique black and white images. They were square-shaped and the sides subtended a visual angle of 3.27° and 2.92° in Experiments 1 and 2, respectively. This difference was because in Experiment 1 a background frame surrounded the figure portion, whereas this was cropped in Experiment 2. We generated two versions of each image by inverting the polarity so that the face portion was either defined by the black or white region. Therefore, there were altogether 18 unique vase-face images.

In Experiment 3, we used the same 18 vase-face images as in Experiment 2, but inverted (Figure 3B). This preserves the low-level feature properties but the outer portion is typically not perceived as faces, especially not for brief stimulus presentations as used here.

In Experiment 4, we used custom-made Navon-type figures (Navon, 1977) to probe local versus global perception. The background portion of the image was a circular area, whose diameter subtended 3.68°. Triangles and squares were arranged into groups that could be interpreted as larger triangles or squares (Figure 3C). All stimuli were incongruent, so the local component shapes were never the same as the global group. There were four configurations, but again in both polarities, so that there were altogether 8 unique images.

In Experiment 5, we used 2 bistable color images on a white background by Octavio Ocampo (Figure 3D) that can either be interpreted as a face or as a picture (specifically, a flower or tree). These are both available online for fair use and are called Family of Birds (https://www.wikiart.org/en/octavio-ocampo/family-of-birds) and Mouth of Flower (https://www.wikiart.org/en/octavio-ocampo/mouth-of-flower). They were square-shaped and the sides subtended 4.9°. We generated two versions of each image by mirroring it horizontally. Thus, there were altogether 4 unique images.

Stimuli in each experiment were scaled to ensure that the task-relevant portion of the image overlapped in each experiment. Based on pilot experiments we also chose the size of stimuli to ensure that participants would be able to perform the task adequately. For example, if the Navon-type figures in Experiment 4 had been too small they would have never been interpreted locally because the local shapes could not be resolved.

Stimuli were presented on a uniform grey background while a blue dot fixation target (diameter: 0.2°) was presented in the screen center. Possible stimulus locations at a given eccentricity were

placed in equal angular steps around an imaginary circle. There were 18 locations in Experiment 1: six were centered 1.96° from fixation and the rest at twice that distance (3.92°). There were 8 locations in Experiments 2-4 centered 3.27° from fixation and in Experiment 5 at a distance of 3.92°. This difference ensured that the task-relevant portion of the flower-face images in Experiment 5 overlapped the same locations as in Experiments 2-4.

Procedure

Participants sat in a darkened room and viewed the stimuli displayed on a computer screen (Samsung 2233RZ, resolution 1680 x 1050, refresh rate 120Hz). Stimuli were pre-generated images and were presented using MATLAB (MathWorks, Inc.) and Psychtoolbox 3 (Brainard, 1997). Participants stabilized their head on a chin rest and were asked to fixate a central target throughout the whole experiment.

Each trial (Figure 2B) began with a fixation period during which only the fixation target was presented on a uniformly grey screen for 500ms in Experiment 1 and 800ms in Experiments 2-5. Then the test stimulus appeared for 200ms at one of the possible locations. After it had disappeared the participants were asked to make their response by pressing one of two buttons on a computer keyboard. The fixation target would slightly increase in size to 0.26° for 100ms to indicate that the program had registered a response.

In Experiments 1, 2, and 5, participants reported if they saw the stimulus as a face or object (vase or plant, respectively). In Experiment 3 they reported whether they saw the inner or the outer portion of the stimulus as figure compared to the background. The outside portion corresponds to the region that is seen as faces in the upright illusion stimulus. In Experiment 4, they reported whether they saw the stimulus as triangles or as squares. This in turn corresponds to whether they interpreted it locally or globally.

In all experiments, trials were divided into three blocks during which participants were given the opportunity to rest. They pressed a button when ready to continue. In each block, all possible stimuli were tested at each of the possible locations. In Experiment 4, all conditions (every combination of unique stimulus and location) were shown twice, and in Experiment 5 four times per block. In the other three experiments each combination was only shown once per block. There were therefore 972 trials (18 stimuli x 18 locations x 3 blocks) overall in Experiment 1, 432 trials (18 stimuli x 8 locations x 3 blocks) in Experiments 2-3; 384 trials (2 repeats x 8 stimuli x 8 locations x 3 blocks) in Experiment 4, and 384 trials (4 repeats x 4 stimuli x 8 locations x 3 blocks) in Experiment 5. The order of stimuli and locations across trials in a block was pseudo-randomized in each session and participant. Due to a technical failure, one participant only completed 240 trials in the first session of Experiment 3. This meant that not all possible stimuli were presented to them in that session. Excluding this participant from the study completely did however not alter the interpretation of the results.

Participants participated in three separate sessions in Experiment 1 and two sessions in Experiments 2-5. These sessions were carried out several days to weeks apart. Usually we aimed to

test participants on successive days but because of scheduling conflicts and availability issues this was not always possible. The time between sessions varied from 1-18 (mean: 4.9) days in Experiment 1 and 1-6 (mean: 2.1) days in Experiments 2-5. Usually Experiments 2-5 were conducted in one sitting per session, but in one case this was not possible due to a scheduling problem.

Statistical analysis

The primary analyses focused on comparing the similarity between spatial patterns in perceptual biases between sessions and conditions. For this purpose, we conducted a second-level analysis. At the first level, we calculated the Pearson correlation across the patterns for each individual participant (qualitatively extremely similar results were found when using Spearman correlation instead). For instance, in Experiment 1 there were 18 stimulus locations and we therefore obtained 18 data points per participants and session, and calculated the within-subject correlation between these 18 points in session 1 and 2. We also conducted complementary analyses where instead of spatial location we compared the similarity of responses to unique stimuli in the experiment.

At the between-subject level, we then tested if the mean correlation across the group was different from zero using a one-sample t-test. We used the Bayes Factor, BF_{10}, to quantify the statistical evidence supporting the alternative (experimental) hypothesis relative to the null hypothesis. This calculation was based on the default Cauchy prior distribution described previously (Rouder et al., 2009), with the standard scale factor of 0.707. This prior is based on typical effect sizes observed in the wider psychology literature but note that qualitatively very similar inferences would be obtained across a broad range of scales. An important feature of Bayesian statistics is that they permit an inference about whether or not the evidence supports the absence of a correlation.

For comparisons between Experiments 2-5, we used the intersession reliability to estimate the maximally achievable correlation. We first extrapolated the reliability of the *n*th experiment, r_n, from its intersession reliability, r_i, using the Spearman-Brown prediction formula (Brown, 1910; Spearman, 1910):

$$r_n = \frac{2r_i}{1 - r_i}$$

We then determined the maximal achievable correlation, r_{ab}, between experiments *a* and *b* using the following equation (Spearman, 1904),

$$r_{ab} = \rho_h \sqrt{r_a r_b}$$

where r_a and r_b are the reliabilities for experiments *a* and *b*, respectively, and ρ_h is the true correlation. We then calculated the percentage of the observed correlation relative to this achievable correlation. We fixed $\rho_h = 1$ to reflect the theoretical possibility that one experiment can completely predict the results of another. This constitutes a conservative *lower bound* of how

much of the explainable variance was in fact explained by the observed correlation. If the true correlation between two experiments were less than 1, this percentage would necessarily be greater than what we report.

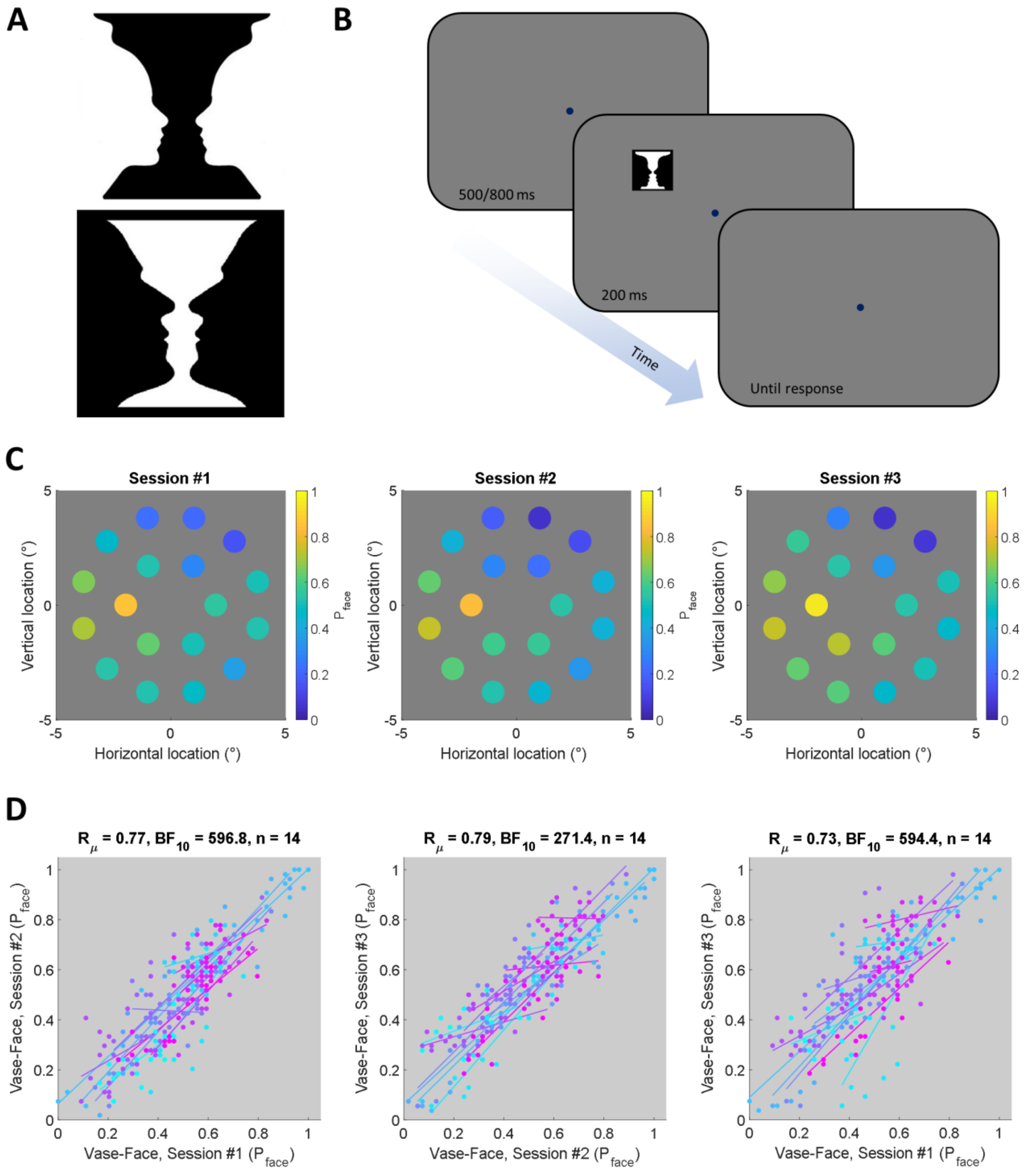

Figure 2. A. Examples of the vase-face illusion. B. Timeline of a typical trial in all experiments (different stimuli were displayed in Experiments 3-5). C. The proportion of trials one participant reported seeing faces (see color code) in each session of Experiment 1 plotted at each visual field location tested. D. Correlation between the three sessions in Experiment 1 across all participants. Dots denote the proportion of trials participants reported seeing faces at a given location. Different colors indicate individual participants. Solid lines show a linear regression between sessions across locations for each participant.

Results

Experiment 1 ("Vase-Face")

We quantified the proportion of trials that 14 individual participants reported seeing a face instead of a vase at 18 locations. Figure 2C shows the stability of patterns of face perception for one participant across separate sessions (see Supplementary Figures S1 and S2 for plot from all 14 individuals). In general, across all participants and stimulus locations the response biases ranged from mostly reporting vases in some locations to most reporting faces in others (Figure 2D). Individuals also evidently differed in terms of the overall response rates: while some participants mostly reported faces, others mostly reported seeing vases. The degree of spatial heterogeneity also varied considerably between participants. Perhaps unsurprisingly, participants with greater variability across locations tended to show stronger intersession reliability, as denoted by the long and steep regression curves parallel to the identity line in Figure 2D.

To quantify the reliability of the within-subject patterns, we calculated the correlation for each participant between sessions (z-transformed), and then determined if the group average r_μ was significantly different from zero (Bayesian one-sample t-test). Figure 2D shows that patterns were strongly correlated between sessions (sessions 1 vs 2: r_μ=0.77, BF_{10}=596.8; sessions 2 vs 3: r_μ=0.79, BF_{10}=271.4; sessions 1 vs 3: r_μ=0.73, BF_{10}=594.4). Peripheral vision is however spatially less precise. These results could therefore have been driven partially by an eccentricity effect. Indeed, averaged across the three sessions and iso-eccentric locations, central stimuli were seen significantly (Bayesian paired t-test: t=3.88, BF_{10}=22.7) more often as faces (P_{faces}=0.59) than peripheral ones (P_{faces}=0.48).

To disentangle potential spatial variation within individual participants from this eccentricity effect, we therefore repeated the same analyses separated by eccentricity. Again, the patterns of biases were very reliable between sessions both for central (sessions 1 vs 2: r_μ=0.7, BF_{10}=117.9; sessions 2 vs 3: r_μ=0.75, BF_{10}=79.6; sessions 1 vs 3: r_μ=0.77, BF_{10}=1205.9) and peripheral stimuli (sessions 1 vs 2: r_μ=0.78, BF_{10}=744.3; sessions 2 vs 3: r_μ=0.77, BF_{10}=120.0; sessions 1 vs 3: r_μ=0.69, BF_{10}=136.1).

Next, we established that these patterns were not similar *across* participants. For this we again used the pattern averaged across the three sessions but now calculated correlations between the patterns for each participant. For 14 participants, this results in 91 unique correlations. Again, we used a Bayesian one-sample t-test to compare if the mean correlation was different from zero. For both the central (r_μ=0.00, BF_{10}=0.116) and peripheral (r_μ=0.01, BF_{10}=0.117) eccentricity the evidence supported the null hypothesis by close to a factor of 10. Therefore, our results suggest that the bias patterns were inconsistent across participants.

We also analyzed how perception varied between the different vase-face images we used. Here, we ignored stimulus location and instead quantified for each of the 18 unique images the proportion of trials for which participants responded to have seen faces. We then calculated a similar second-level analysis as before but for stimuli rather than locations. This revealed a pronounced effect of the stimuli. The intersession correlations were all very strong (all r_μ>0.83, all BF_{10}>393.3). Part of this effect was driven by contrast polarity. On average across the group, participants perceived images in which the inner portion (the "vase") was black on a white background only 40% as faces, while for the white-on-black polarity this was 63% (Bayesian paired t-test: BF_{10}=47.8). However, when splitting the data by stimulus polarity, the intersession correlation remained very strong (black-on-white: all r_μ>0.79, all BF_{10}>1853.2; white-on-black: all r_μ>0.7, all BF_{10}>90.5). This demonstrates that observers varied in terms of how they perceived the unique images irrespective for polarity.

We also again calculated between-subject correlations for the 91 unique pairs of participants to test the consistency of these patterns across participants. Unlike for stimulus position, the pattern of responses to the different stimuli was similar across participants, both for black-on-white images (r_μ=0.49, BF_{10}>>10,000, n=91) and white-on-black images (r_μ=0.56, BF_{10}>>10,000, n=91). Thus, while the spatial heterogeneity of responses was highly idiosyncratic, the effect of individual stimuli was very consistent across participants.

Experiment 2 ("Vase-Face replication")

We then carried out additional experiments to probe potential mechanisms for this perceptual heterogeneity. First, we replicated the biases for face perception as in Experiment 1 in a new experiment but with only 8 possible stimulus locations, all at the same eccentricity. Again, the pattern of biases was reliable across the two test sessions (Supplementary Figures S3A), albeit somewhat less robustly than in Experiment 1 (r_μ=0.49, BF_{10}=10.5, n=19), possibly due to the broader pool of participants and the wider age range.

Note that in this and especially some of the following experiments, a few individuals exhibited ceiling or floor effects, that is, biases at all stimulus locations were identical. This precluded calculating the within-subject correlation for these participants and they were therefore automatically excluded from further statistical analysis (as indicated by t-tests where the sample size is smaller than 20). For example, for the test of intersession reliability one participant was removed because they exhibited no spatial heterogeneity in the second session. This participant generally reported seeing faces almost exclusively at every location. While their bias pattern is doubtless extremely reliable, this prevents any further analysis of spatial heterogeneity.

We also again compared the responses to the 18 unique images, irrespective of spatial location. As in Experiment 1, the intersession correlation was very strong (r_μ=0.81, BF_{10}=6383.2, n=19, one participant was removed for the aforementioned reasons). We also replicated the effect of contrast polarity. On average, participants tended to perceive black-on-white images as faces only on 40% of trials while they did so for white-on-black on 72% of trials (Bayesian paired t-test: BF_{10}=88.9, n=20).

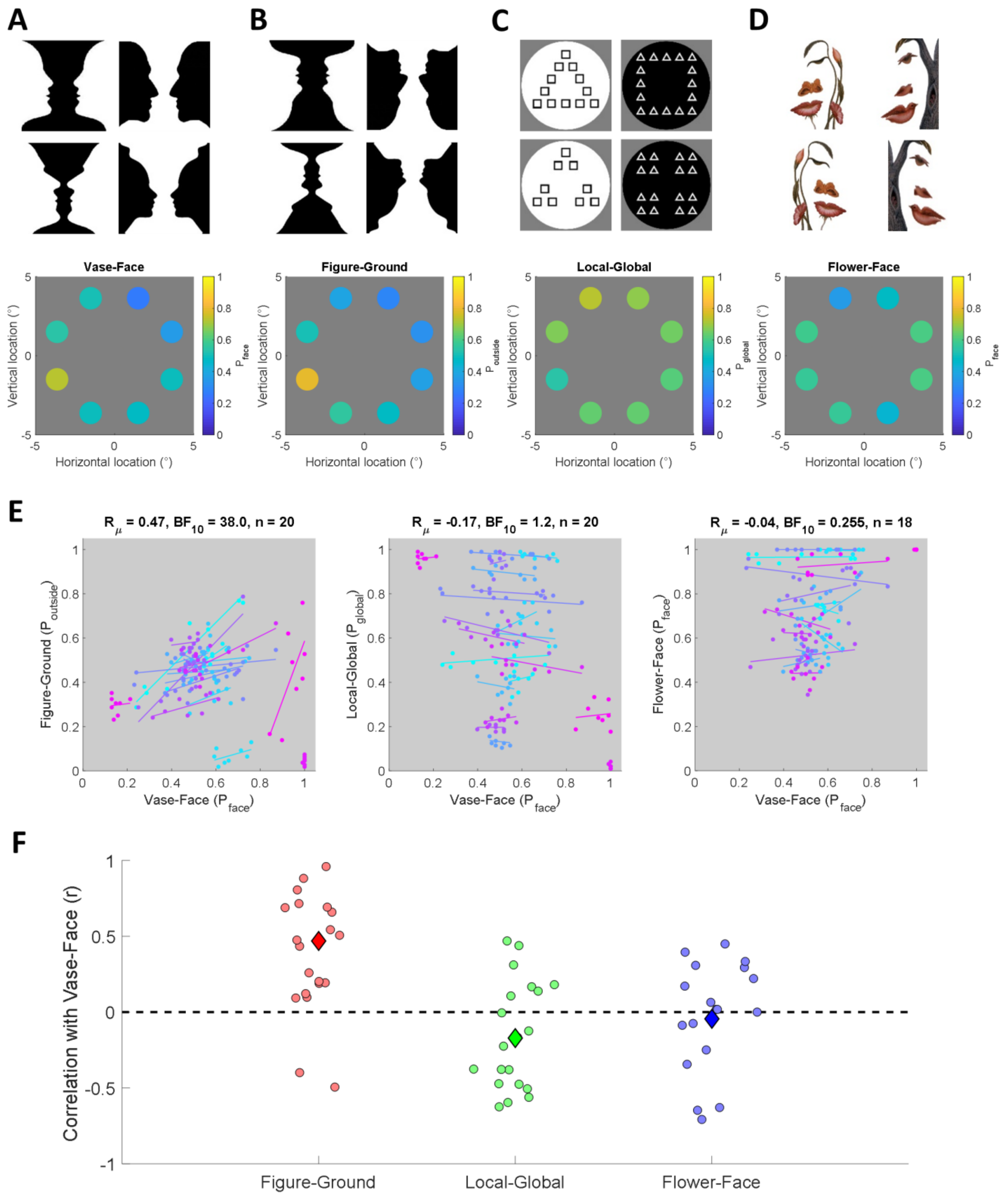

Figure 3. A-D. Example stimuli (top row) and results from one example participant (bottom row) in Experiments 2-5, that is, the vase-face replication (A), the figure-ground (B), local-global (C), and flower-face (D) experiments. Plots show the proportion of trials (see color code) at each visual field location that one participant reported, respectively, seeing as faces (A,D), the outside part of inverted vase-face images as figure (B), or the global configuration in Navon-like stimuli (C). E. Correlation between bias patterns in Experiments 3-5 and Experiment 2 across all participants. Dots denote the proportion of trials participants reported seeing the relevant perceptual state (see above). Different colors indicate individual participants. Solid lines show a linear regression between sessions across locations for each participant. F. Correlation coefficients for comparing patterns in Experiments 3-5 with those in Experiment 2. Each dot denotes results from one participant. The larger diamond symbols indicate the group mean correlation r_μ.

Experiment 3 ("Figure-Ground")

We then tested if similar spatial heterogeneity was observed with inverted vase-face stimuli. Inversion typically disrupts face perception and recognition substantially (Valentine, 1988; Yin, 1969). Accordingly, our participants informally reported not seeing faces in these inverted images. Therefore, if bias patterns were similar between Experiments 2 and 3, this would suggest these two experiments share a common figure-ground segregation process, rather than being specifically related to faces. Instead, in this experiment we asked participants to report whether they saw the inner or outer portion of the image as the figure. Interpreting the outer portion as figure is equivalent to seeing faces in the upright vase-face image.

In general, response biases varied considerably across participants and stimulus locations, although there was no location for which any participant only reported seeing the outside portion of the image as figure. The same participant who almost exclusively reported seeing faces in Experiment 2 also showed very little spatial heterogeneity in Experiment 3. They generally almost never reported seeing the outside portion of the image as the figure, and in the second session never reported such a percept. They were therefore automatically removed from the analysis of intersession reliability.

Participants also varied in terms of their overall response rates. Bias patterns were relatively reliable across sessions, albeit much less so than for upright vase-face images (r_{μ}=0.37, BF_{10}=5.6, n=19, one participant was removed for the aforementioned reasons). This could be due to a reduction of the spatial heterogeneity from session 1 and 2, as illustrated by the flat slopes for some of the individual regression curves in Supplementary Figure S3B.

Nevertheless, the biases for vase-face perception in Experiment 2 were well correlated (r_{μ}=0.47, BF_{10}=38, n=20) with those for perceiving the outside portion as the figure in Experiment 3 (Figure 3E, left panel, 3F). The single participant who exhibited minimal response bias in Experiments 2 and 3, did not follow this general pattern. In Experiment 2, they almost always perceived faces, but in Experiment 3 they almost never reported the outside portion of the image as the figure. In the left panel of Figure 3E their data cluster in the bottom right corner.

Based on the intersession reliability for the two experiments, we can estimate the maximally achievable correlation between them to be r=0.60. The correlation we actually observed was approximately 82% of that, indicating there was a strong relationship between them. This suggests that a similar process governs figure-ground interpretation for upright and inverted vase-face images.

Next, we compared the response rates between the 18 unique images, irrespective of stimulus location. For these analyses, we again must remove the participant who showed minimal response bias. For the analysis of intersession reliability another participant was also removed. Due to a technical problem they did not complete all trials in the first session. This meant that not all stimuli were presented to this participant and therefore no correlation could be computed (see Methods).

Again, we found a strong intersession correlation (r_μ=0.55, BF_{10}=20.9, n=18), suggesting that the responses to the images were temporally reliable. But interestingly, unlike for upright vase-face images in Experiments 1 and 2, there was little evidence of a polarity effect in terms of how often participants reported seeing faces and results instead supported the null hypothesis (black-on-white=0.39; white-on-black=0.44; BF_{10}=0.274, n=19). Comparing the pattern of responses in Experiments 2 and 3, we found inconclusive evidence for a modest correlation (r_μ=0.44, BF_{10}=0.768, n=19). Since these two experiments used identical images (upright and inverted, respectively) this speaks against a strong stimulus effect being shared across those experiments.

Experiment 4 ("Local-Global")

Next, we tested whether this spatial heterogeneity was due to variability in a general perceptual organization process. We probed whether individuals showed variability across the visual field as to whether they interpreted Navon-type stimuli locally as small triangles/squares, or globally as coherent groups arranged into a triangle or square, respectively. While there was some variability across the visual field, the degree of heterogeneity was low (Figure 3E, middle panel). Rather, individuals tended to report similar levels of local and global perception across all locations. This can be seen in the flat slopes of the individual regression curves and minimal dispersion for each participant along the Y axis.

Bias patterns were therefore only moderately correlated between sessions (Supplementary Figure S3), and the statistical evidence was subtly in favor of the null hypothesis (r_μ=0.21, BF_{10}=0.781, n=20), although this level of evidence is inconclusive. Interestingly, when ignoring spatial patterns the mean response rates for each participant correlated strongly between sessions (Bayesian correlation test, (Wetzels & Wagenmakers, 2012): r=0.81, BF_{10}=1838.3, n=20). These results suggest that overall observers have robust preferences for the local or global interpretation across sessions, but spatial patterns for this local-global task are not reliable.

Importantly, bias patterns for seeing faces in Experiment 2 were only weakly correlated with the patterns of interpreting stimuli globally versus locally in Experiment 4 (Figure 3E,F; r_μ=-0.17, BF_{10}=1.2). While this level of evidence is inconclusive, it suggests that only a small fraction of the variance across spatial locations is shared between those experiments. The maximally achievable correlation for this comparison was 0.48. Our actual observed (negative) correlation was only about 35% of this.

As for previous experiments, we also compared the response pattern between the 8 unique stimuli, irrespective of location. The average intersession reliability was actually negative (r_μ=-0.44, BF_{10}=1.7, n=20). While there was only inconclusive statistical evidence for this negative correlation, this confirms that participants did not consistently interpret a given image locally or globally.

Experiment 5 ("Flower-Face")

Experiment 3 already suggested that the spatial heterogeneity in vase-face perception relates to how individuals segregate images into figure and background rather than reflecting face-

processing. Nevertheless, we wanted to test further if these biases could be due to face processing. We therefore used a different set of bistable images that can also be perceived as a face or an object (tree or flower). These were not classical figure-ground stimuli. Rather, to see a face in these images, one must interpret most of the image, including parts of the background, as belonging to the face. To see the plants/trees and birds the observer must focus on some of the finer detail. If the biases in Experiments 2 were generally related to face perception, then there should be a positive correlation between the bias patterns in Experiments 2 and 5.

Again, the pattern of face biases was relative reliable across sessions, supported by modest statistical evidence (r_{μ}=0.40, BF_{10}=4.3, n=16). However, four participants showed little to no evidence of spatial heterogeneity and (almost) only reported seeing faces. They were therefore removed from further analysis of spatial heterogeneity. In general, participants tended to see the images more frequently as faces than objects, as revealed by the clustering of data points in the upper right quadrant in Supplementary Figure S3D. Some participants also showed a reduction of spatial heterogeneity from session 1 to 2, as shown by the flat regression curves.

Critically, the correlation between face biases in Experiment 2 and 5 was only very weak, and statistical evidence supported the null hypothesis (r_{μ}=-0.04, BF_{10}=0.255, n=18). Notably, five participants mostly reported seeing the images as faces irrespective of location, suggesting that these individuals did not experience strong bistability for these images at all. Two participants must be removed because they almost exclusively reported seeing faces in either experiment, which precluded any further analysis of the correlation between them. The maximally achievable correlation for this comparison was 0.61, and the actual observed correlation only about 7% of that.

Finally, we again compared response rates for the 4 unique images, irrespective of location. This revealed compelling evidence for a strong intersession correlation (r_{μ}=0.94, BF_{10}=98.7, n=16). Participants were therefore very consistent in whether they interpreted these images as faces or objects.

Discussion

We probed spatial heterogeneity in the perception of bistable images. First, we established that individual perception of Rubin's vase-face illusion depends on retinal location. The pattern of these perceptual biases across the visual field were reliable across several days to weeks. This suggests relatively stable mechanisms underlie these biases.

One such factor was common across participants: stimuli closer to fixation were perceived more often as faces than at more peripheral locations. This mirrors the suggestion that face processing is biased towards central vision and reflects an eccentricity gradient in the organization of ventral stream areas (Uri Hasson et al., 2002). A simpler explanation could however be that this difference is related to poorer visual acuity in the periphery.

Critically, eccentricity alone cannot explain the perceptual heterogeneity we observed. The bias patterns for only central or more peripheral locations were also highly reliable but showed little correlation between individual observers. As such, these patterns constitute unique "perceptual fingerprints". Our findings thus parallel the spatial heterogeneity reported for perceiving stimulus size (Afraz et al., 2010; Moutsiana et al., 2016; Schwarzkopf & Rees, 2013), location (Kosovicheva & Whitney, 2017), orientation, shape, and complex features like the apparent identity, age, or gender of faces (Afraz et al., 2010; Visconti di Oleggio Castello et al., 2018) and ambiguous motion (Wexler et al., 2015). The findings are also reminiscent of heterogeneity in the perception of bistable motion stimuli depending on their orientation (Wexler, 2018).

Interestingly, the perception of vase-face images also strongly depended on contrast polarity. Participants tended to perceive images in which the inner portion (the "vase") was black on a white background as vases, and vice versa for the opposite polarity. However, this was specific for upright vase-face images. No such polarity effect was observed for inverted images. There was also little evidence of a consistent response pattern across the 18 unique vase-face images. Nevertheless, both for upright and inverted vase-face images the response rates generally depended strongly on the stimulus. The tendency to interpret a given image as faces or a vase was reliable across sessions and very consistent between participants. This contrasts with the results for spatial heterogeneity where the evidence strongly suggested that – beyond the difference between central and peripheral eccentricities – the bias patterns were unique to each observer. Note that these stimulus differences cannot explain the spatial heterogeneity we found: every unique stimulus was presented multiple times at each possible location, and the order of stimuli and locations was randomized between sessions and participants.

Some previous research posited that such idiosyncratic heterogeneity could result from undersampling (Afraz et al., 2010; de Haas et al., 2016; Kosovicheva & Whitney, 2017; Moutsiana et al., 2016). If the neurons whose receptive fields cover a particular visual field location are selective for a particular stimulus feature (position, shape, or a class of objects, like faces) then this would lead to a biased stimulus encoding that could skew perceptual interpretation (Figure 1). Related research also reported spatial heterogeneity for processing facial features. Observers are more accurate when discriminating images of eyes shown in the upper versus the lower visual field, and vice versa for mouths (de Haas et al., 2016). Observers tend to fixate the nose and thus eyes and mouths would typically appear in the upper and lower hemifields, respectively. The heterogeneity could therefore represent fine-tuning of the visual system based on experience: it would be more economical to predominantly encode a given feature in visual field locations where it is most likely to appear.

Could such undersampling explain the biases we observed for the vase-face illusion? We further compared biases for vase-face perception with those for inverted vase-face images. Inversion disrupts face processing (Valentine, 1988; Yin, 1969) and our participants did not report seeing faces in these inverted images. Yet we found that patterns for interpreting the outside of the inverted images as the figure were strongly correlated with the patterns for seeing faces in the

upright illusion stimuli. The outside portion of these images is the equivalent of the faces in the upright image. This suggests that the spatial biases we observed may not be related to face processing *per se*, but rather more generally to figure-ground segregation.

It could be argued that perceiving faces in the vase-face images requires more local processing of fine spatial detail. Interpreting the shape in the image as a profile view of a face entails identifying the individual features of the figure boundary as nose, forehead, eye sockets, etc. In contrast, this is not necessary for perceiving vases – one could easily perceive a vase without any of the spatial detail in the image at all. Therefore, our next experiment explicitly tested whether the results for upright and inverted vase-face images could relate to a propensity for local processing at a given stimulus location. We used stimuli that can be either interpreted based on the local components or the global group (Navon, 1977). Locations where individuals perceived faces were not strongly related to local or global processing. While the statistical evidence for this null result was inconclusive, spatial patterns in this task were also less reliable across sessions than in the other experiments. Interestingly, unlike the spatial heterogeneity, the *overall* rates for each participant averaged across locations were very reliable between sessions. This points to inter-individual differences in cognitive style across observers. Some individuals tend to process these images more locally than others, but this was independent of stimulus location. Of course, we cannot rule out that a different local-global task might exhibit spatial heterogeneity, and that this correlates with the perception of our vase-face images.

Lastly, we also compared the vase-face illusion to the perception of different bistable images. These images could also be perceived as faces or as a different complex object (tree or flower). If the heterogeneity we observed for the vase-face illusion were due to undersampling of the visual field by neurons generally sensitive to faces, then the bias patterns for these images should be similar. We found that they were not, providing further evidence that these spatial biases may not be related to face processing. Most participants in the experiment also generally tended to see these images as faces. This also speaks against an undersampled receptive field mosaic of neurons tuned to faces and objects, respectively. Yet, as for the local-global task, we cannot rule out that different ambiguous stimuli that allow a face interpretation would exhibit greater spatial heterogeneity and that this variability in turn correlates with the perception of vase-face images.

Importantly, how participants interpreted vase-face images at a given visual field location was similar for upright and inverted images. They did however not interpret inverted images as faces. Conversely, observers did not perceive the flower-face images as faces at the same locations. Rather than undersampling by face-sensitive neurons, this therefore points towards heterogeneity in figure-ground segregation. Because bias patterns were inconsistent between most experiments, our findings rule out a simple response bias by which participants would simply respond in a particular way at a given spatial location.

What neural mechanism could explain such biases? The notion of neurons tuned to particular figure-ground interpretations that undersample the visual field seems implausible. Instead, our findings could reflect whether the mosaic of receptive fields at that location undersamples the

inner or outer portion of the image. If the inner portion of the image is encoded more precisely in a particular location, it may be more likely to be interpreted as figure. Alternatively, we cannot rule out entirely that the inverted vase-face images still drive face-sensitive neurons. If these neurons are selective only to profile views of faces, they would not respond to the frontal faces in the flower-face stimuli. As a result, the bias patterns in these two experiments would differ. A third, and perhaps the most parsimonious alternative, is that the biases we observed are due to the spatial sensitivity at each location. Perceiving a face in the vase-face images may require distinguishing finer spatial detail than detecting a vase. This could be achieved by focusing more on the local detail compared to the global whole, but our results from the local-global experiment speak against this idea: individuals were not more likely to perceive faces in locations where they tended to report the local features of Navon-type stimuli.

The perception of faces could be directly linked to spatial sensitivity. We already observed that images in more peripheral locations, where spatial sensitivity is reduced, tended to be less likely to be perceived as faces. Our previous research has demonstrated that spatial sensitivity varies across the visual field, even at the same eccentricity, and could result in heterogeneity of perceptual biases (Moutsiana et al., 2016). Therefore, it is possible that locations where we tend to perceive faces – or the outer portion of the image as the figure – are also those were spatial vision is more precise. This could be tested by future research.

Conclusion

Our findings show that an observer’s perception of vase-face images varies reliably across the visual field. Some of this heterogeneity is shared across observers, but there are also unique patterns that constitute idiosyncratic perceptual fingerprints. This intra-individual variability is probably not due to undersampling of the visual field by face-responsive neurons or due to variations in local vs global processing. Rather, a more parsimonious explanation is that this heterogeneity results from spatial variations in lower-level visual function.

Data availability

The raw data, stimulus materials, and code for this study are publicly available at osf.io/ap8me

Acknowledgments

This work was supported by a European Research Council Starting Grant to DSS (310829-WMOSPOTWU).

References

Afraz, A., Pashkam, M. V., & Cavanagh, P. (2010). Spatial heterogeneity in the perception of face and form attributes. *Current Biology: CB*, *20*(23), 2112–2116. https://doi.org/10.1016/j.cub.2010.11.017

Anstis, S. (1998). Picturing peripheral acuity. *Perception*, *27*(7), 817–825.

Bedell, H. E., & Johnson, C. A. (1984). The perceived size of targets in the peripheral and central visual fields. *Ophthalmic & Physiological Optics: The Journal of the British College of Ophthalmic Opticians (Optometrists)*, *4*(2), 123–131.

Brainard, D. H. (1997). The Psychophysics Toolbox. *Spatial Vision*, *10*(4), 433–436.

Brown, W. (1910). Some Experimental Results in the Correlation of Mental Abilities1. *British Journal of Psychology, 1904-1920*, *3*(3), 296–322. https://doi.org/10.1111/j.2044-8295.1910.tb00207.x

de Haas, B., Schwarzkopf, D. S., Alvarez, I., Lawson, R. P., Henriksson, L., Kriegeskorte, N., & Rees, G. (2016). Perception and Processing of Faces in the Human Brain Is Tuned to Typical Feature Locations. *The Journal of Neuroscience: The Official Journal of the Society for Neuroscience*, *36*(36), 9289–9302. https://doi.org/10.1523/JNEUROSCI.4131-14.2016

Duncan, R. O., & Boynton, G. M. (2003). Cortical magnification within human primary visual cortex correlates with acuity thresholds. *Neuron*, *38*(4), 659–671.

Groen, I. I. A., Silson, E. H., & Baker, C. I. (2017). Contributions of low- and high-level properties to neural processing of visual scenes in the human brain. *Philosophical Transactions of the Royal Society of London. Series B, Biological Sciences*, *372*(1714). https://doi.org/10.1098/rstb.2016.0102

Hasson, U., Hendler, T., Ben Bashat, D., & Malach, R. (2001). Vase or face? A neural correlate of shape-selective grouping processes in the human brain. *Journal of Cognitive Neuroscience*, *13*(6), 744–753. https://doi.org/10.1162/08989290152541412

Hasson, Uri, Levy, I., Behrmann, M., Hendler, T., & Malach, R. (2002). Eccentricity bias as an organizing principle for human high-order object areas. *Neuron*, *34*(3), 479–490. https://doi.org/10.1016/s0896-6273(02)00662-1

Helmholtz, H. (1867). *Handbuch der Physiologischen Optik*.

Hsu, S.-M., & Young, A. (2004). Adaptation effects in facial expression recognition. *Visual Cognition*, *11*(7), 871–899. https://doi.org/10.1080/13506280444000030

Kay, K. N., Weiner, K. S., & Grill-Spector, K. (2015). Attention reduces spatial uncertainty in human ventral temporal cortex. *Current Biology: CB*, *25*(5), 595–600. https://doi.org/10.1016/j.cub.2014.12.050

Kosovicheva, A., & Whitney, D. (2017). Stable individual signatures in object localization. *Current Biology: CB*, *27*(14), R700–R701. https://doi.org/10.1016/j.cub.2017.06.001

Moutsiana, C., de Haas, B., Papageorgiou, A., van Dijk, J. A., Balraj, A., Greenwood, J. A., & Schwarzkopf, D. S. (2016). Cortical idiosyncrasies predict the perception of object size. *Nature Communications*, *7*, 12110. https://doi.org/10.1038/ncomms12110

Navon, D. (1977). Forest before trees: The precedence of global features in visual perception. *Cognitive Psychology*, *9*(3), 353–383. https://doi.org/10.1016/0010-0285(77)90012-3

Newsome, L. R. (1972). Visual angle and apparent size of objects in peripheral vision. *Perception & Psychophysics*, *12*(3), 300–304. https://doi.org/10.3758/BF03207209

O'Craven, K. M., Downing, P. E., & Kanwisher, N. (1999). FMRI evidence for objects as the units of attentional selection. *Nature*, *401*(6753), 584–587. https://doi.org/10.1038/44134

Peatfield, N., Mueller, N., Ruhnau, P., & Weisz, N. (2015). Rubin-vase illusion perception is predicted by prestimulus activity and connectivity. *Journal of Vision*, *15*(12), 429. https://doi.org/10.1167/15.12.429

Pelli, D. G. (1997). The VideoToolbox software for visual psychophysics: Transforming numbers into movies. *Spatial Vision*, *10*(4), 437–442.

Rouder, J. N., Speckman, P. L., Sun, D., Morey, R. D., & Iverson, G. (2009). Bayesian t tests for accepting and rejecting the null hypothesis. *Psychonomic Bulletin & Review*, *16*(2), 225–237. https://doi.org/10.3758/PBR.16.2.225

Schwarzkopf, D. S., & Rees, G. (2013). Subjective size perception depends on central visual cortical magnification in human v1. *PloS One*, *8*(3), e60550. https://doi.org/10.1371/journal.pone.0060550

Schwarzkopf, D. S., Song, C., & Rees, G. (2011). The surface area of human V1 predicts the subjective experience of object size. *Nature Neuroscience*, *14*(1), 28–30. https://doi.org/10.1038/nn.2706

Schweinberger, S. R., Zäske, R., Walther, C., Golle, J., Kovács, G., & Wiese, H. (2010). Young without plastic surgery: Perceptual adaptation to the age of female and male faces. *Vision Research*, *50*(23), 2570–2576. https://doi.org/10.1016/j.visres.2010.08.017

Spearman, C. (1904). The Proof and Measurement of Association between Two Things. *The American Journal of Psychology*, *15*(1), 72–101. https://doi.org/10.2307/1412159

Spearman, C. (1910). Correlation Calculated from Faulty Data. *British Journal of Psychology, 1904-1920*, *3*(3), 271–295. https://doi.org/10.1111/j.2044-8295.1910.tb00206.x

Storrs, K. R., & Arnold, D. H. (2012). Not all face aftereffects are equal. *Vision Research*, *64*, 7–16. https://doi.org/10.1016/j.visres.2012.04.020

Valentine, T. (1988). Upside-down faces: A review of the effect of inversion upon face recognition. *British Journal of Psychology (London, England: 1953)*, *79 (Pt 4)*, 471–491. https://doi.org/10.1111/j.2044-8295.1988.tb02747.x

Visconti di Oleggio Castello, M., Taylor, M., Cavanagh, P., & Gobbini, M. I. (2018). Idiosyncratic, Retinotopic Bias in Face Identification Modulated by Familiarity. *ENeuro*, *5*(5), 1–14. https://doi.org/10.1523/ENEURO.0054-18.2018

Wetzels, R., & Wagenmakers, E.-J. (2012). A default Bayesian hypothesis test for correlations and partial correlations. *Psychonomic Bulletin & Review*, *19*(6), 1057–1064. https://doi.org/10.3758/s13423-012-0295-x

Wexler, M. (2018). Multidimensional internal dynamics underlying the perception of motion. *Journal of Vision*, *18*(5), 7. https://doi.org/10.1167/18.5.7

Wexler, M., Duyck, M., & Mamassian, P. (2015). Persistent states in vision break universality and time invariance. *Proceedings of the National Academy of Sciences of the United States of America*, *112*(48), 14990–14995. https://doi.org/10.1073/pnas.1508847112

Yin, R. K. (1969). Looking at upside-down faces. *Journal of Experimental Psychology*, *81*(1), 141–145. https://doi.org/10.1037/h0027474
